# Biomechanical evaluation of multi-rod constructs to stabilize an S1 pedicle subtraction osteotomy (PSO): a finite element analysis

**DOI:** 10.1007/s43390-023-00784-w

**Published:** 2023-11-30

**Authors:** Niloufar Shekouhi, Sudharshan Tripathi, Vijay K. Goel, Alekos A. Theologis

**Affiliations:** 1 Engineering Center for Orthopedic Research Excellence (E-CORE), Departments of Bioengineering and Orthopaedic Surgery, University of Toledo, Toledo, OH USA; 2 Department of Orthopaedic Surgery, University of California-San Francisco (UCSF), 500 Parnassus Ave, MUW 3rd Floor, San Francisco, CA 94143 USA

**Keywords:** Finite element analysis, Pedicle subtraction osteotomy, Sacral osteotomy, Multi-rod constructs, Rod stresses

## Abstract

**Purpose:**

To develop and validate a finite element (FE) model of a sacral pedicle subtraction osteotomy (S1-PSO) and to compare biomechanical properties of various multi-rod configurations to stabilize S1-PSOs.

**Methods:**

A previously validated FE spinopelvic model was used to develop a 30° PSO at the sacrum. Five multi-rod techniques spanning the S1-PSO were made using 4 iliac screws and a variety of primary rods (PR) and accessory rods (AR; lateral: Lat-AR or medial: Med-AR). All constructs, except one, utilized a horizontal rod (HR) connecting the iliac bolts to which PRs and Med-ARs were connected. Lat-ARs were connected to proximal iliac bolts. The simulation was performed in two steps with the acetabula fixed. For each model, PSO ROM and maximum stress on the PRs, ARs, and HRs were recorded and compared. The maximum stress on the L5–S1 disc and the PSO forces were captured and compared.

**Results:**

Highest PSO ROMs were observed for 4-Rods (HR + 2 Med-AR). Constructs consisting of 5-Rods (HR + 2 Lat-ARs + 1 Med-AR) and 6-Rods (HR + 2 Lat-AR + 2 Med-AR) had the lowest PSO ROM. The least stress on the primary rods was seen with 6-Rods, followed by 5-Rods and 4-Rods (HR + 2 Lat-ARs). Lowest PSO forces and lowest L5–S1 disc stresses were observed for 4-Rod (Lat-AR), 5-Rod, and 6-Rod constructs, while 4-Rods (HR + Med-AR) had the highest.

**Conclusion:**

In this first FE analysis of an S1-PSO, the 4-Rod construct (HR + Med-AR) created the least rigid environment and highest PSO forces anteriorly. While 5- and 6-Rods created the stiffest constructs and lowest stresses on the primary rods, it also jeopardized load transfer to the anterior column, which may not be favorable for healing anteriorly. A balance between the construct’s rigidity and anterior load sharing is essential.

**Supplementary Information:**

The online version contains supplementary material available at 10.1007/s43390-023-00784-w.

## Introduction

Sagittal spinal malalignment is an important driver of functional disability that may develop secondary to a variety of causes, including de novo spinal degeneration, neuromuscular disorders, iatrogenic flatback, and traumatic and/or pathological fractures [1–5]. While surgical management aimed to improve lumbar lordosis (LL) through the lumbar spine itself that restores lumbopelvic harmony, pelvic tilt, and global sagittal balance results in satisfactory outcomes for the vast majority of patients, there exist a small subset of patients in whom restoration of LL is not adequate, as they have pathologically high pelvic incidences (PI) as the driver of deformity. Pathologically high PIs are most commonly secondary to lumbosacral kyphosis from high-grade spondylolisthesis, prior fusions, and/or sacral fractures with residual sacral kyphosis [6–8]. These deformities require a surgical strategy in the form of a pedicle subtraction osteotomy (PSO) through the first sacral segment (S1), which reduces the PI itself [8–13].

The major advantage of an S1-PSO is that it directly decreases a patient’s pelvic incidence, which allows one to directly address a deformity at its apex in patients with pathologically high PIs. In contrast to lumbar PSOs, S1-PSOs are considerably less common operations owing to their challenging surgical demands, high complication profile, and relatively limited indications [12]. However, similar to lumbar PSOs, S1-PSOs require robust stability consisting of multi-rod constructs to optimize durability of the operation (i.e., maintain deformity correction and decrease rod fractures) [14–23]. While these constructs have been evaluated comprehensively for lumbar PSOs, there is a paucity of information and comparative data on surgical stabilization strategies for S1-PSOs. As such, the goals of this study were to develop and validate a finite element analysis (FEA) model of an S1-PSO and to assess and compare the biomechanical properties of various multi-rod configurations to stabilize S1-PSOs.

## Materials and methods

In this study, a previously validated osseoligamentous 3D spinopelvic model (T10-pelvis) was used to develop a 30° PSO in the first sacral segment [24, 25]. The intact model was reconstructed from computed tomography (CT) scans of the human spine using MIMICS (Materialize Inc., Leuven, Belgium) software. IAFE-MESH (University of Iowa, Iowa) and HyperMesh (Altair Engineering, Michigan, USA) were used to create hexahedral elements (C3D8) of the vertebrae and tetrahedral elements (C3D4) of the pelvis, respectively. The meshed components were assembled in Abaqus 6.14 (DassaultSystemes, Simile Inc., Providence, RI, USA). The spinal and sacroiliac ligaments were modeled using truss elements. In the vertebral body, a layer of 0.5 mm cortical bone was simulated to surround the cancellous bone.

The intervertebral discs were composed of annulus fibrosus and nucleus pulposus. Annulus fibrosis was simulated using a solid ground substance (C3D8 elements) reinforced with rebar elements (embedded at 30 angles). The sacroiliac joint was modeled using a soft contact with exponential behavior. The material properties were adapted from the literature and assigned to each component (Table 1) [24, 26].

**Table 1:** Material properties used in model development adapted from literature [24, 26]

Components	Element formulation	Young’s modulus (MPa)/Poisson’s ratio
Vertebral cortical bone	Isotropic, elastic hex elements (C3D8)	12,000/0.3
Vertebral cancellous bone	Isotropic, elastic hex elements (C3D8)	100/0.2
Pelvic cortical bone	Isotropic, elastic hex elements (C3D8)	17,000/0.3
Pelvic cancellous bone	Isotropic, elastic hex elements (C3D8)	100/0.2
Annulus (ground)	Neo-Hookean, hex elements (C3D8)	C10 = 0.348, D1 = 0.3
Annulus (fiber)	Isotropic, elastic hex elements (C3D8)	6/0.45
Nucleus	Isotropic, elastic hex elements (C3D8)	1/0.4999
Apophyseal joints	Nonlinear soft contact, GAPUNI elements	–
Sacroiliac joints	Nonlinear soft contact	–
Ligaments	Hypo-elastic, tension only, Truss elements (T3D2)	Nonlinear stress − strain curves
Ti6Al4V Pedicle screws/horizontal rods	Isotropic, tetrahedron elements (C3D4)	105,000/0.36
CoCr Primary and accessory rods	Isotropic, tetrahedron elements (C3D4)	241,000/0.3

### S1-PSO model development

**Fig. 1:**
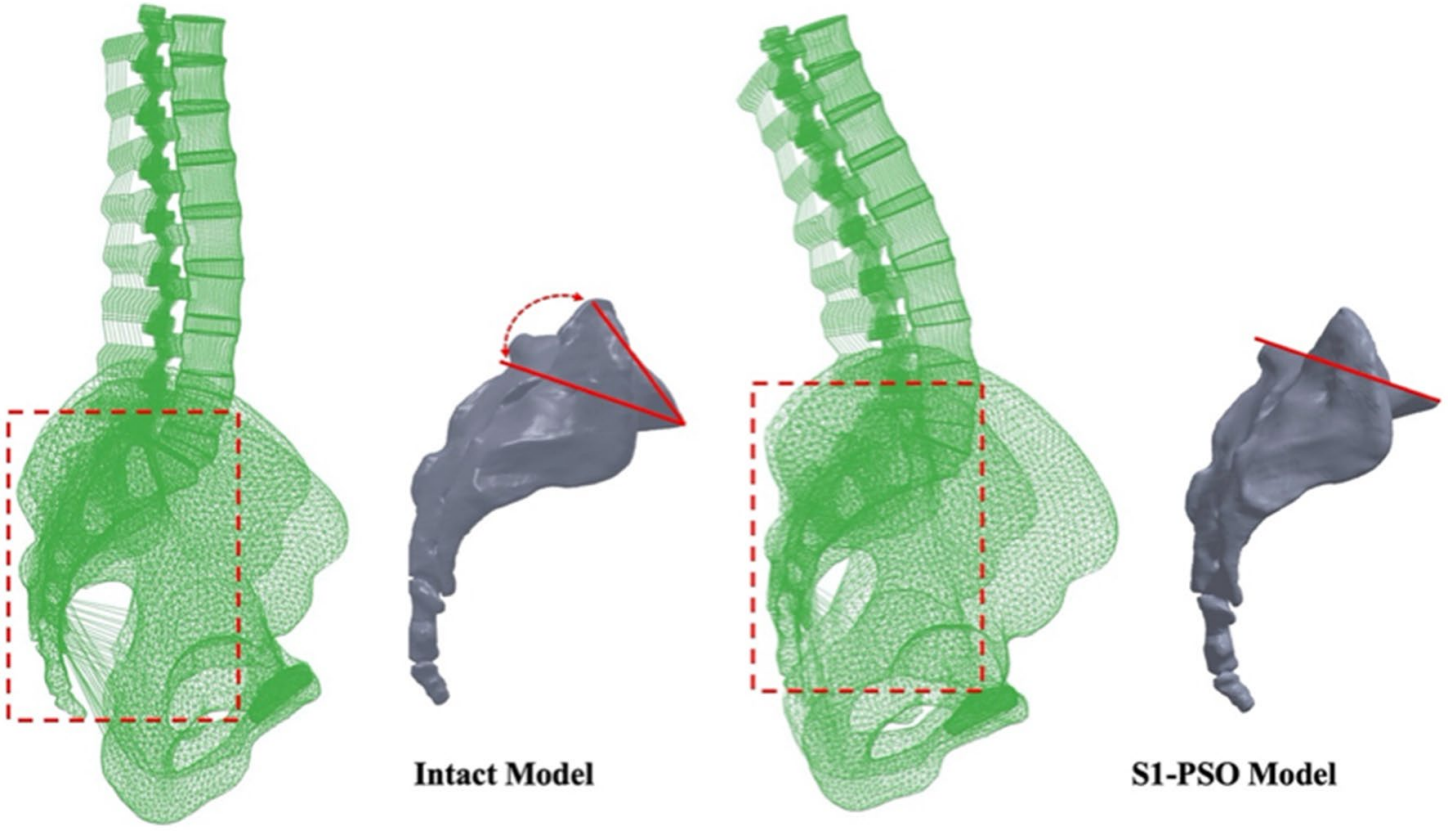
Schematic of the intact and S1-PSO models. The intact unmeshed sacrum was used to resect a 30° wedge-shaped structure through the first sacral segment

The intact unmeshed sacrum was imported into SolidWorks to resect a 30° wedge-shaped structure (Fig. 1). This part was imported into HyperMesh (Altair Engineering, Michigan, USA) to create the 6-node linear triangular elements (C3D6). Given that the intact model was previously validated, we used the same mesh type and seed size as the sacrum in the intact model. The sacrum was then imported into Abaqus and merged with the pelvis in an intact model. The same material properties were defined as intact alignment (a trabecular core bone surrounded by a 1 mm cortical layer). Truss elements were employed to reconstruct the ligamentous tissue at the sacroiliac joint as well as the sacrotuberous and sacrospinous ligaments.

**Fig. 2:**
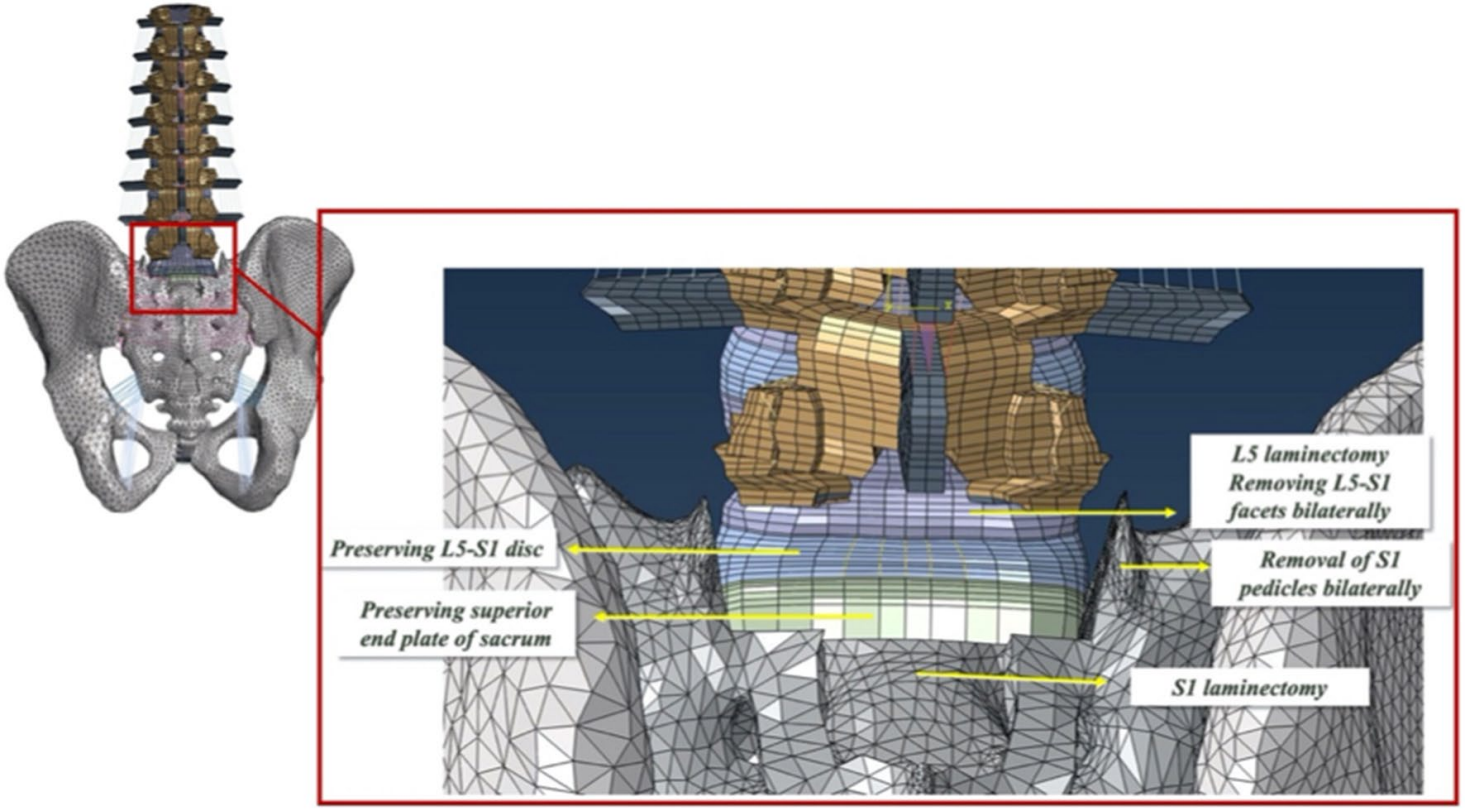
A posterior view of the S1-PSO model. The L5–S1 facets and S1 pedicles were removed bilaterally, followed by a complete L5 and S1 laminectomy. The L5–S1 intervertebral disc and the superior end plate of the sacrum were preserved

The L5–S1 facets and S1 pedicles were removed bilaterally, followed by complete L5 and S1 laminectomies. The L5–S1 intervertebral disc and superior endplate of the sacrum were preserved (Fig. 2).

### Model instrumentation

**Fig. 3:**
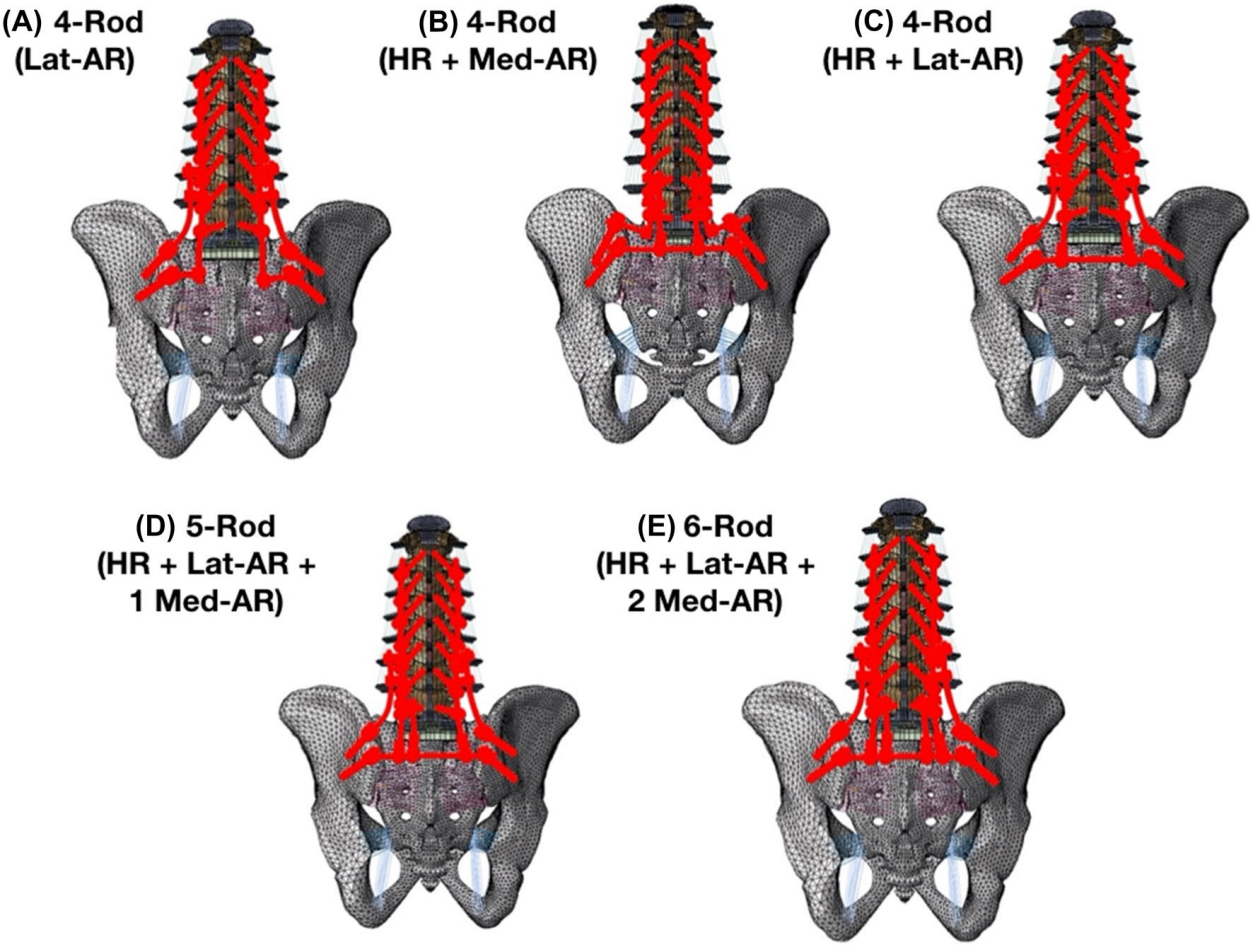
Schematic drawings of the five multi-rod configurations: **A** 4-Rod (2 Lateral Accessory Rods); **B** 4-Rod (Horizontal Rod + 2 Medial Accessory Rods); **C** 4-Rod (Horizontal Rod + 2 Lateral Accessory Rods); **D** 5-Rod (Horizontal Rod + 2 Lateral Accessory Rods + 1 Medial Accessory Rod); **E** 6-Rod (Horizontal Rod + 2 Lateral Accessory Rods + 2 Medial Accessory Rod). *HR* horizontal rod, *Lat-AR* lateral accessory rod, *Med-AR* medial accessory rod

Model instrumentation [including pedicle screws, iliac screws, rods, lateral connectors, in-line/ “domino” connectors, and rod/rod connectors (open up/open up: “W”)] was designed in SolidWorks (Dassault Systèmes SolidWorks Corporation, Waltham, MA, USA). All models included instrumentation from T11 to the pelvis (Fig. 3). Two iliac bolts were placed in each hemipelvis (4 total). All pedicles, starting from T11 to the pelvis, were instrumented bilaterally using titanium alloy polyaxial screws. The size and length of the pedicle screws were consistent in all models. Polyaxial screws were simulated using a methodology presented in the literature [14, 27]. The primary (PR), accessory (AR), and horizontal rods (HR) were tied to the tulip in all the models. A tie constraint was used to secure the rods to all connectors (“W”, in-line/ “domino”, and lateral connectors). All accessory rods and primary rods were 5.5 mm cobalt–chrome and all horizontal rods were 5.5 mm titanium alloy (Table 1) [24, 26].

The anterior section was tied to the osteotomy site, and a surface-to-surface interaction (friction = 0.46) was defined between the two resected segments at the posterior site. Seed sizes and mesh types were assigned based on our preliminary mesh convergence study. The following 5 multi-rod configurations were created and simulated:

*(1) 4-Rod (Lat-AR)* Two primary rods and two lateral accessory rods. The primary rods (T11 to pelvis) were connected to the most distal of the two iliac screws via a lateral connector. The accessory rods were attached to the most proximal of the two iliac bolts and to the primary rods laterally via W-connectors (positioned between L2–L3 and L3–L4 segments). This model did not include a horizontal rod.

*(2) 4-Rod (HR* + *Med-AR)* Two primary rods, a horizontal rod (HR), and two medial accessory rods (Med-AR). The primary rods (T11 to pelvis) were connected to the most proximal iliac screws via lateral connectors. The HR was connected to the most distal iliac bolts in each hemipelvis. The accessory rods were connected perpendicular to the HR by connecting them to an in-line/ “domino” rod–rod connector and a lateral connector (Fig. 3). The ARs were secured to the primary rods medially via W-connectors (positioned between L3–L4 and L4–L5 segments).

*(3) 4-Rod (HR* + *Lat-AR)* Two primary rods, a horizontal rod, and two lateral accessory rods. The HR was connected to the most distal iliac bolts in each hemipelvis. The primary rods were connected perpendicular to the HR by connecting them to an in-line/ “domino” rod–rod connector and a lateral connector (Fig. 3). The accessory rods were attached to the most proximal of the two iliac bolts and to the primary rods laterally via W-connectors (positioned between L2–L3 and L3–L4 segments).

*(4) 5-Rod (HR + Lat-AR + 1 Med-AR)* This construct is a modification of the 4-Rod (HR + Lat-AR) construct with one additional medial accessory rod, which is connected perpendicular to the HR via an in-line/ “domino” rod–rod connector and a lateral connector (Fig. 3) and medial to the left primary rod via a W-connector at L4–L5 so as to span the S1 PSO site (Fig. 3).

**Fig. 4:**
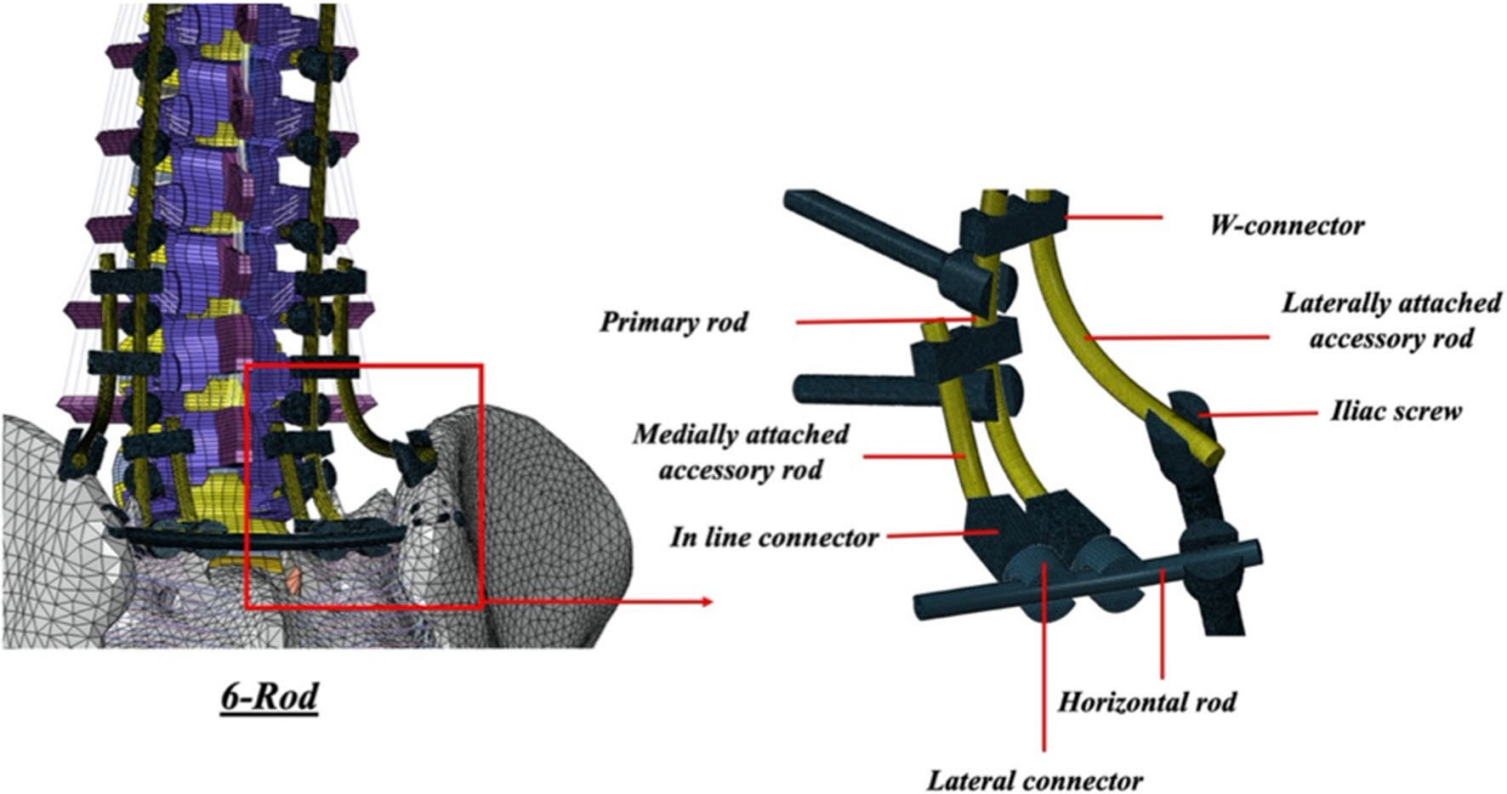
Schematic of 6-Rod configuration demonstrating attachment techniques of the primary and horizontal rod to the horizontal rods. Primary rods and medial accessory rods were attached to the horizontal rod via a combination of in-line (“domino”) and lateral connectors. Lateral accessory rods were secured to the primary rods laterally via rod–rod (open up/open up) “W” connectors

*(5) 6-Rod (HR + Lat-AR + 2 Med-AR)* This construct is a modification of the 4-Rod (HR + Lat-AR) construct with two additional medial accessory rods, which are each connected perpendicular to the HR via an in-line/ “domino” rod–rod connector and a lateral connector (Fig. 4) and medial to the primary rods via W-connectors at L4–L5 so as to span the S1 PSO site (Figs. 3, 4).

### Loading and boundary conditions

Loading for the PSO spinopelvic FEA model was applied in two steps. In step 1, 300 N was applied to the thoracic spine, 400 N to the lumbar spine, and 400 N to the sacrum using the follower load technique [24, 25]. In Step 2, pure moments of 7.5 Nm were applied to the top endplate of the T10 vertebral body in all three anatomical directions. During the simulation, the acetabular surfaces of pelvis were fixed at all degrees of freedom.

### Data analysis

Following each simulation step, the PSO ranges of motion (ROM) were calculated. For each configuration, the maximum stress magnitude on the PRs was recorded, and the percentage differences in the AR stresses with respect to the PR’s stresses were calculated. Furthermore, the maximum von Mises stress on the L5–S1 annulus fibrosis and the force across the PSO site were captured and compared for all models.

## Results

### Model validation

The validation graphs for the compression stiffness of the intact and S1-PSO models are shown in Supplementary Fig. 1. Stiffness showed a reduction from 79.3 N/mm at baseline to 42.4 N/mm following S1-PSO which was in agreement with the *in vitro* study of *Vanaclocha *et al*.* [26]. FE predictions for the intact fell within their range, and S1-PSO showed 7% lower stiffness than their results. However, the normalized stiffness obtained in our study [(S1-PSO/Intact) × 100] was similar to that reported by *Vanaclocha *et al*.* [26]. Therefore, the model was validated.

### PSO range of motions (Fig. 5)

**Fig. 5:**
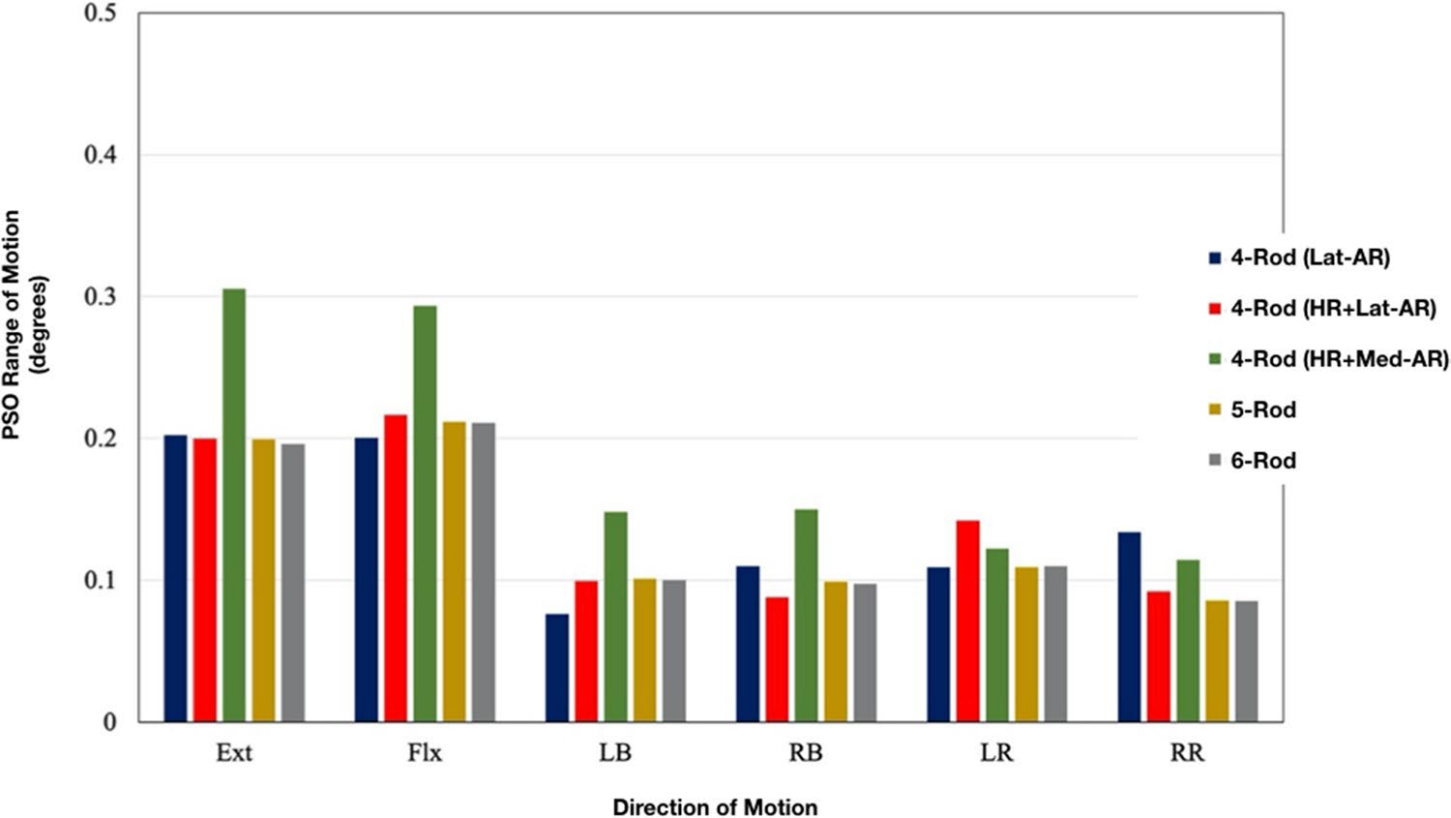
Range of motions across the S1-PSO for each multi-rod construct

The greatest PSO motion in all directions was observed in the 4-Rod (HR + Med-AR) construct. Relative to this model, PSO motion was decreased by the following percentages for each of the other four constructs: − 49% [4-Rod (Lat-AR)], − 41% [4-Rod (HR + Lat-AR)], − 35% (5-Rods), and − 36% (6-Rods). In both left and right axial rotations, all three of the 4-Rod constructs demonstrated a higher PSO ROM than the 5-Rod and 6-Rod configurations.

### Stresses on primary and accessory rods (Table 2)

**Table 2:** Maximum von Mises stresses on primary rods and accessory rods

	4-Rod (Lat-AR)	4-Rod (HR + Med-AR)	4-Rod (HR + Lat-AR)	5-Rod	6-Rod
Ext					
PR	175.8 MPa	139.8 MPa	83.5 MPa	91.4 MPa	77.6 MPa
Med-AR	n/a	78.7 (− 43.7%)	n/a	61.7 (− 32.5%)	60.5 (− 22.0%)
Lat-AR	202.7 (+ 15.3%)	n/a	182.1 (+ 118.0%)	169.7 (+ 85.7%)	171.5 (+ 121.0%)
Flx					
PR	255.8 MPa	267.2 MPa	184.9 MPa	186.2 MPa	150.9 MPa
Med-AR	n/a	106.3 (− 60.2%)	n/a	59.3 (− 68.2%)	58.7 (− 61.1%)
Lat-AR	159.8 (− 37.5%)	n/a	173.3 (− 6.3%)	178.0 (− 4.4%)	176.7 (− 17.1%)
LB					
PR	219.9 MPa	231.3 MPa	147.7 MPa	141.6 MPa	121.0 MPa
Med-AR	n/a	120.5 (− 40.1%)	n/a	54.5 (− 61.5%)	54.1 (− 55.3%)
Lat-AR	217.6 (− 1.0%)	n/a	176.4 (+ 19.4%)	183.4 (+ 29.5%)	186.3 (+ 54.0%)
RB					
PR	215.4 MPa	184.1 MPa	136.0 MPa	118.2 MPa	109.3 MPa
Med-AR	n/a	154.2 (− 16.2%)	n/a	66.4 (− 43.8%)	65.5 (− 40.1%)
Lat-AR	233.1 (+ 8.2%)	n/a	209.4 (+ 54.0%)	197.9 (+ 67.4%)	199.8 (+ 82.8%)
LR					
PR	212.4 MPa	263 MPa	157.4 MPa	147.5 MPa	150.6 MPa
Med-AR	n/a	118.5 (− 54.9%)	n/a	88.1 (− 40.3%)	87.5 (− 41.9%)
Lat-AR	191.8 (− 9.7%)	n/a	165.1 (+ 4.9%)	164.6 (+ 11.6%)	158.3 (+ 5.1%)
RR					
PR	261.1 MPa	182.3 MPa	129.5 MPa	154.2 MPa	130.0 MPa
Med-AR	n/a	154.4 (− 15.3%)	n/a	42.5 (− 72.4%)	42.0 (− 67.7%)
Lat-AR	169.2 (− 35.2%)	n/a	172.0 (+ 32.8%)	165.4 (+ 7.3%)	165.1 (+ 27.0%)

Percentages for Med-AR and Lat-AR represent relative change compared to primary rod stresses

*Ext* extension, *Flx* flexion, *LB* left lateral bending, *RB* right lateral bending, *LR* left axial rotation, *RR* right axial rotation, *Lat-AR* lateral accessory rod, *HR* horizontal rod, *Med-AR* medial accessory rod

Among all the models, the 6-Rod configuration showed the least stress on the primary rods. Relative to this model, von Mises stresses on the primary rods were increased by the following percentages for each of the other 4 constructs: + 127% [4-Rod (Lat-AR)], + 91% [4-Rod (HR + Med-AR)], + 24% [4-Rod (HR + Lat-AR)], and + 23% (5-Rods).

The FE predictions indicated that Lat-AR had higher von Mises stresses compared to PRs, while Med-AR had lower von Mises stresses compared to the PRs for all constructs.

### PSO forces (Table 3)

**Table 3:** Force across the osteotomy site for each configuration

	4-Rod (Lat-AR)	4-Rod (HR + Med-AR)	4-Rod (HR + Lat-AR)	5-Rod	6-Rod
Ext	149.7	168.2	160.2	154.6	152
Flx	265.2	280.7	302.6	265.5	261.9
LB	218.7	224	237.7	209.8	206.7
RB	198.9	224.1	223.1	207.7	204.5
LR	204.7	229.6	228.3	211.8	208.4
RR	212	222.1	229.8	208.2	205.1

*Ext* extension, *Flx* flexion, *LB* left lateral bending, *RB* right lateral bending, *LR* left axial rotation, *RR* right axial rotation, *Lat-AR* lateral accessory rod, *HR* horizontal rod, *Med-AR* medial accessory rod

In all directions, the 6-Rod, 4-Rod (Lat-AR), and 5-Rod configurations had the lowest PSO forces. In contrast, the 4-Rod (HR + Lat-AR) and 4-Rod (HR + Med-AR) had the highest PSO forces in all motions.

### Von Mises stresses on L5–S1 Disc (Fig. 6)

**Fig. 6:**
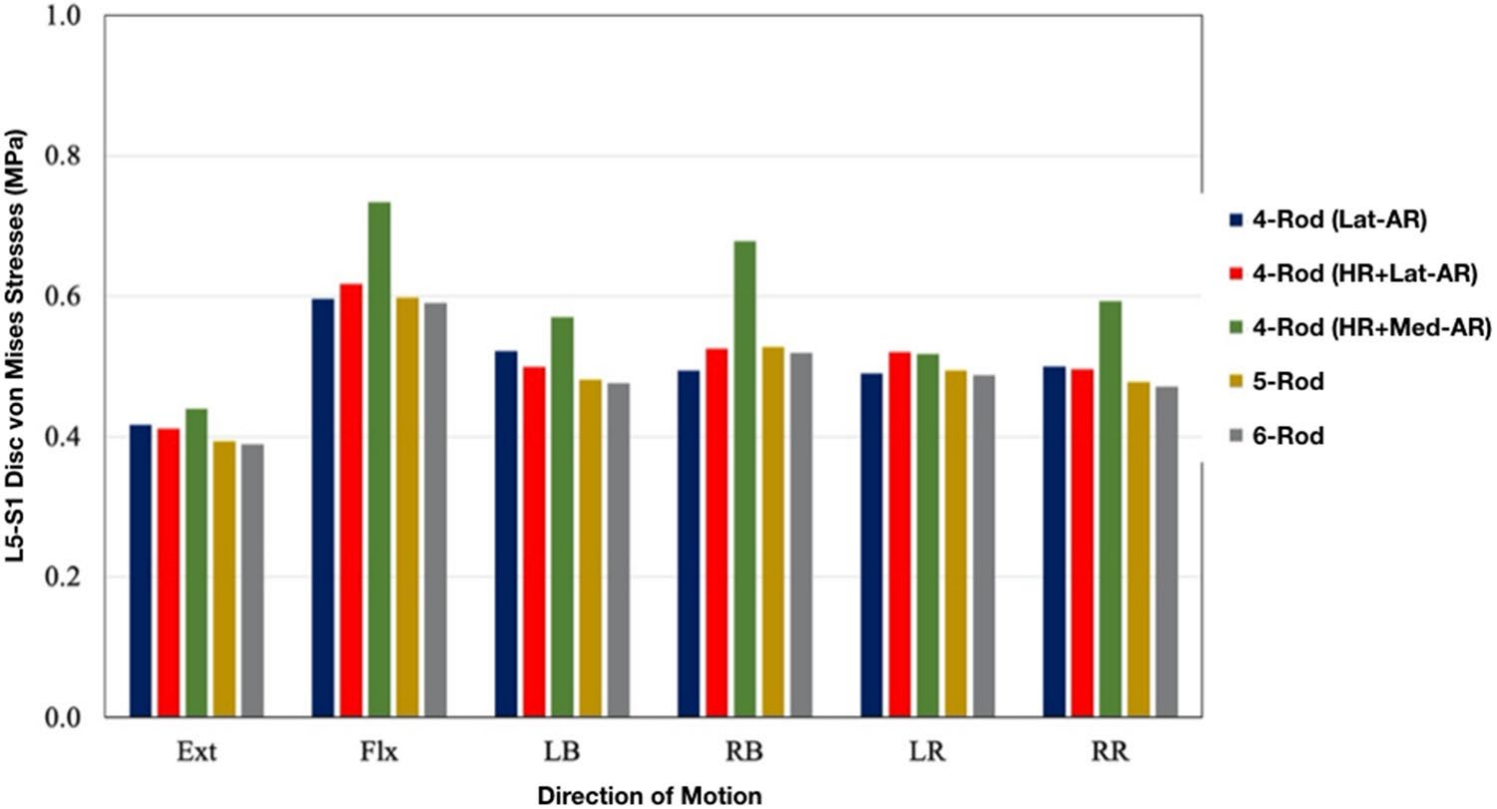
Von Mises stresses on the L5–S1 annulus fibrosis for each multi-rod construct

The lowest von Mises stresses on the L5–S1 disc were observed for the 5- and 6-Rod constructs. Compared to 6-Rods, von Mises stresses on the L5–S1 disc’s annulus fibrosis were greater by the following percentages for each of the 4-rod constructs: + 10% [4-Rod (Lat-AR)], + 7% [4-Rod (HR + Lat-AR)], and + 31% [4-Rod (HR + Med-AR)]. Compared to 6-Rods, 5-Rods showed similar von Mises stresses on the annulus fibrosis (within 1–2% difference).

## Discussion

A closing wedge 3-column osteotomy through the first sacral segment (i.e., S1-PSO) is a powerful surgical technique to decrease pelvic incidence magnitude and improve sagittal balance from lumbosacral deformities and kyphosis. Given it is infrequently indicated and performed, results of S1-PSO’s long-term clinical outcomes and complication profiles are limited to several case reports and small cohort studies [8–13]. As there is no known prior investigation comparing various surgical strategies to stabilize these challenging 3-column osteotomies, we developed and validated an FEA model of an S1-PSO and assessed and compared the biomechanical properties of various multi-rod configurations to stabilize S1-PSOs. There major findings were as follows: (1) a 4-Rod construct consisting of a horizontal rod and 2 medial accessory rods (HR + Med-AR) had the greatest ROM across the PSO site, the highest von Mises stresses across the L5–S1 disc, and the highest forces across the PSO site; (2) stresses in the primary rods for this 4-Rod (HR + Med-AR) construct were second highest, and (3) 5-Rod and 6-Rod constructs had the least ROM across the PSO site, the lowest stresses on the primary rods, the lowest von Mises stresses across the L5–S1 disc, and the lowest forces across the PSO site. Importantly, the S1-PSO model created in this study was validated.

The first biomechanical evaluation of an S1-PSO was performed by *Vanaclocha *et al*.* [26]. Their cadaver testing showed that LL and PT increased, whereas PI and SS decreased [26]. Consistent with the study of *Vanaclocha *et al*.*, we found that LL increased, PI and SS decreased, and compression stiffness reduced following an S1-PSO (Supplementary Fig. 1).

Numerous studies have demonstrated that multi-rod constructs across lumbar PSOs provide more robust biomechanical environments and decrease rod fracture fractures relatively to 2-rod constructs [14–23]. Creating multi-rod constructs across S1-PSOs can be more challenging given the relatively limited space/anatomy as well as limited available osseous fixation points distal to S1 (i.e., in the sacrum and pelvis). Additionally, achieving fusion across the lumbosacral junction can be quite challenging. As such, multi-rod constructs across the lumbosacral junction, especially for S1 PSOs, are important for achieving stability to prevent loss of deformity correction and for decreasing the chances of rod fractures across the PSO site. To create a solid foundation distal to an S1-PSO, placement of dual iliac screws in each hemipelvis is commonly needed and recommended, as it provides greater stability than single iliac screw fixation [28, 29]. From these four points of iliac fixation, one may design a variety of rod configurations that cross the S1 osteotomy site. We chose three constructs that included 4 total rods cross the S1-PSO site and 2 constructs in which there were > 4 rods crossing the PSO site (5-Rod and 6-Rod; i.e., “super multi-rod constructs”). To make the 5-Rod and 6-Rod constructs (given only 4 iliac screw attachment points), we utilized a rod that ran horizontal (medial–lateral) over the sacrum and attached to the two most distal iliac screws. This horizontal rod was also used to create two of the three 4-Rod constructs. Attachments of this horizontal rod to primary and/or accessory rods that run perpendicular to it and run cranial–caudal across the PSO site were accomplished by first connecting a lateral connector to the horizontal rod, then attaching one end of an in-line/ “domino” rod–rod connector to the rod portion of the lateral connector, and then connecting the accessory rod to the other end of the in-line/ “domino” rod–rod connector (Fig. 4). This “cross-bar” technique was adopted from instrumentation strategies used to improve rotation stability following sacrectomies [30, 31]. While this horizontal rod/cross-bar did not itself appear to provide any biomechanics advantages or disadvantages in our study, it can be a useful method by which one can create multi-rod constructs across an S1-PSO (Fig. 4).

When comparing the 4-Rod constructs, our FEA indicated that the 4-Rod configuration consisting of a horizontal rod and 2 medial accessory rods (HR + Med-AR) had the greatest ROM across the PSO site, the highest von Mises stress across the L5–S1 disc, and the highest forces across the PSO site. These same findings were the case when comparing the 4-Rod (HR + Med-AR) to the 5- and 6-Rods. Thus, this construct demonstrated the least rigidity and, subsequently, a higher load was carried by the anterior column, which is postulated to promote bone healing and fusion at the osteotomy site anteriorly and decrease the chances of non-union [32]. The exact reason for this 4-Rod construct’s biomechanical behavior is not entirely clear; however, it might be due to the fact that this was the only configuration in which the primary rods were attached to the more proximal iliac screws; whereas in all other models, the primary rods were secured to the most distal iliac screws either through lateral connectors [4-Rod (Lat-AR)] or via connections to the horizontal rods [i.e., 4-Rod (HR + Lat-AR), 5-Rods, and 6-Rods]. Of note, the 4-Rod (HR + Med-AR) model showed the lowest stress on the horizontal rod, which is another potential advantage of this configuration.

When evaluating the impact of the 5- and 6-Rod techniques, our data demonstrated that both techniques created the most rigid environments across the PSO site (lowest PSO ROMs and lowest von Mises stress in the L5–S1 discs). This subsequently resulted in the lowest PSO forces as well as the lowest stress on the primary rods. While these data may suggest a lower chance of rod failure of the primary rods, stress shielding by the posterior instrumentation and offloading of the anterior column may not provide an optimal healing environment across the PSO site anteriorly [32]. This is similar to a recent study by *Shekouhi *et al*.* in which 5-Rod and 6-Rod constructs across a lumbar PSO created the most rigid environment, but decreased PSO forces, relative to 4-Rod constructs [15]. This potential issue with anterior loading may be countered by addition of an anterior fusion. Also of note is that higher stresses were observed on the horizontal rods in the 5- and 6-Rod configurations owing to the presence of multiple interconnecting components and their stress intensification effect (a combination of in-line and lateral connectors).

The results of this study should be considered within the context of these limitations. While we believe that the accuracy of this FEA model is acceptable, simulation being performed with no muscle forces and using uncomplicated geometries of the implants and simplified contact and constraints may all jeopardize its accuracy in simulating the forces during an S1-PSO. Additionally, interconnections of the rods, screws, lateral connectors, and anatomy were all in ideal conditions, and residual stresses secondary to screw/rod tightening and rod contouring were not considered. Furthermore, the model’s results may be influenced by other factors, including rod diameter, material, and bend magnitude. Additionally, as it has been previously demonstrated that the four different Roussouly type’s sagittal alignments have different kinetic and biomechanical responses under various loading conditions and that lumbar lordosis has strong positive correlations with posterior rod strains [33, 34], it is possible that the results of our study may also be influenced and changed by different shapes of the lumbar spine including variations in apex of lordosis, segmental and regional lumbar lordosis, pelvic tilt, and PI. Future studies would benefit from assessing the biomechanics of S1-PSOs for different Roussouly types that incorporate varying segmental and regional lumbar lordosis, pelvic tilts, and pelvic incidences. While we did not include interbody support at L5–S1 so as to tease out the relative contributions of the different posterior instrumentation techniques, it is possible that our observed biomechanical differences may be different if interbody support was performed at L5–S1. Furthermore, the use of S2 alar–iliac screws (S2AI) were not investigated in this study. The use of S2AI screws could be utilized to recreate the same 4-rod instrumentation constructs in models A and B if one pelvic bolt and one S2AI screw were used in each hemipelvis. The S2AI screws in these 4-rod scenarios would replace the most distal pelvic screw so as to allow them to be connected to the primary rods directly and to allow the lateral accessory rods to connect to the more proximal pelvic bolts (model A) and to each other via a horizontal rod (model B). The use of S2AI screw, however, would not be able to be utilized to recreate the same 5-Rod and 6-Rod constructs or the third 4-rod construct (model C), as they would prevent concomitant attachments to the horizontal rods and the primary rods. While we assume that the biomechanics of the 4-Rod constructs using S2AI screws would be similar to our findings using 2 pelvic bolts, we are unable to provide quantitative data to confirm this assumption, as the use of S2AI screws was beyond the scope of this study. Nevertheless, future studies utilizing S2AI screws would be highly beneficial. Another arena that was beyond the scope of our study was testing different S1 PSO angles (i.e., 10° vs. 20° vs. 30°). We focused on a 30-degree osteotomy angle for the PSO, as it was felt to represent the worst-case scenario given greater bone resection and anticipated greater associated instability. Reducing the osteotomy angle may result in changes to the biomechanical environment. Specifically, smaller angles may potentially result in lower ROM, less stress on the rods, and lower L5–S1 disc stresses. However, it is also likely that the differences would not be linear or directly proportional to the angle of osteotomy give the highly complex interplay between the angle of correction, patient anatomy (lordosis, pelvic tilt, pelvic incidence), and instrumentation constructs. As such, future studies would benefit from quantifying the biomechanical differences between varying S1 PSO angles to refine our understanding of optimal patient-specific treatment. Finally, because the margin of important difference and the exact margin of error in our observed absolute values are not known, we are not able to comment upon the clinical and biomechanical significances of our observed biomechanical differences and relative clinical performances of our different constructs. Despite these limitations, the results of this study may be considered a unique additional to the limited, but growing, literature on this distinctive 3-column osteotomy, as this is the first comparative biomechanical analysis of different multi-rod constructs to stabilize an S1-PSO.

## Conclusion

In this first FE analysis of an S1-PSO, the 4-Rod construct (HR + Med-AR) created the least rigid environment and highest PSO forces anteriorly. While 5- and 6-Rods created the stiffest constructs and lowest stresses on the primary rods, it also jeopardized load transfer to the anterior column, which may not be favorable for healing anteriorly. A balance between the construct’s rigidity and anterior load sharing is essential. Additional in vitro and clinical investigations would be beneficial to confirm these findings.

### Supplementary Information

Below is the link to the electronic supplementary material.

Supplementary file1 (JPEG 104 KB)
