# Supplementary figures and images for: Biomechanical evaluation of multi-rod constructs to stabilize an S1 pedicle subtraction osteotomy (PSO): a finite element analysis
Source: Spine Deform. 2023 Nov 30;12(2):313–22. doi: 10.1007/s43390-023-00784-w (PMC10866773; doi:10.1007/s43390-023-00784-w)

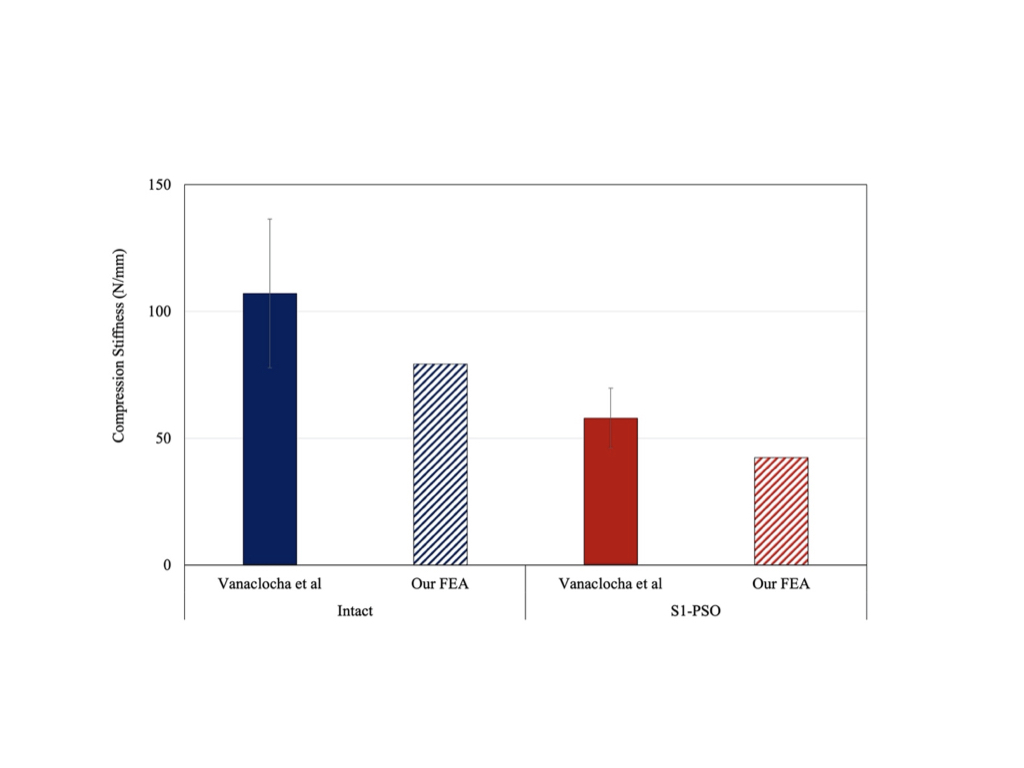

Supplement: Supplementary file 1 — Supplementary file1 (JPEG 104 KB) [file 43390_2023_784_MOESM1_ESM.jpg]
